# Diet Reconstruction and Resource Partitioning of a Caribbean Marine Mesopredator Using Stable Isotope Bayesian Modelling

**DOI:** 10.1371/journal.pone.0079560

**Published:** 2013-11-13

**Authors:** Alexander Tilley, Juliana López-Angarita, John R. Turner

**Affiliations:** 1 School of Ocean Sciences, Bangor University, Menai Bridge, Anglesey, United Kingdom; 2 Talking Oceans Foundation, Bogotá DC, Colombia; 3 Environment Department, University of York, York, United Kingdom; National Institute of Water & Atmospheric Research, New Zealand

## Abstract

The trophic ecology of epibenthic mesopredators is not well understood in terms of prey partitioning with sympatric elasmobranchs or their effects on prey communities, yet the importance of omnivores in community trophic dynamics is being increasingly realised. This study used stable isotope analysis of ^15^N and ^13^C to model diet composition of wild southern stingrays *Dasyatis americana* and compare trophic niche space to nurse sharks *Ginglymostoma cirratum* and Caribbean reef sharks *Carcharhinus perezi* on Glovers Reef Atoll, Belize. Bayesian stable isotope mixing models were used to investigate prey choice as well as viable Diet-Tissue Discrimination Factors for use with stingrays. Stingray δ^15^N values showed the greatest variation and a positive relationship with size, with an isotopic niche width approximately twice that of sympatric species. Shark species exhibited comparatively restricted δ^15^N values and greater δ^13^C variation, with very little overlap of stingray niche space. Mixing models suggest bivalves and annelids are proportionally more important prey in the stingray diet than crustaceans and teleosts at Glovers Reef, in contrast to all but one published diet study using stomach contents from other locations. Incorporating gut contents information from the literature, we suggest diet-tissue discrimination factors values of Δ^15^N ≊ 2.7‰ and Δ^13^C ≊ 0.9‰ for stingrays in the absence of validation experiments. The wide trophic niche and lower trophic level exhibited by stingrays compared to sympatric sharks supports their putative role as important base stabilisers in benthic systems, with the potential to absorb trophic perturbations through numerous opportunistic prey interactions.

## Introduction

Understanding the trophic niche and ecological role of elasmobranchs in community structure is of crucial management and conservation importance [1], [2]. It is becoming increasingly evident that omnivores provide stability to food webs [3]. Natural systems representing a mix of strong and weak trophic interactions, are said to be resistant to trophic perturbations and cascade [4], [5]. However, in marine systems subject to selective fishing pressure, the community-wide impacts of fishing are stronger than expected because fishing preferentially targets strongly interacting apex predator species whose removal can destabilize the food web [5]. The likelihood of trophic cascades occurring after the depletion of these strong interactors will thus depend on the relative fraction of strong omnivory [5]. In light of global shark population declines, the trophic ecology of marine mesopredators is becoming rapidly more pertinent, as predation (or a release from it) can significantly affect community structure [6], [7].

Stingrays have received scant attention despite their position in marine systems as important omnivorous mesopredators, structuring sediments and communities [8], [9]. Demersal stingrays feed on a range of epibenthic prey including crustaceans, molluscs and fish, and infauna such as polychaetes and sipunculids [10], [11]. Traditionally diet composition has involved the invasive, often lethal process of gut contents analysis, yet a common criticism of this technique is its over-representation of hard-to-digest prey items, such as those with exoskeletons [12], and subsequent underestimation of soft-bodied prey in diet composition. The less invasive analysis of stable isotopes in tissues, is now a widely used method of assessing food web interactions [13]–[15]. Due to the close link between the isotopic ratios of consumers and their prey, Stable Isotope Analysis (SIA) provides a means of reconstructing diet composition and quantifying the ecological niche an animal occupies within its trophic system [16], allowing for more robust diet composition analysis over time, compared to the snapshot sampling of stomach contents.

The composition of stable isotopes (commonly ^13^C and ^15^N) in tissues changes relatively predictably as elements cycle through ecosystems, as a result of the trophic interactions of species [14]. By calculating the area of 2D space occupied on bionomic axes of δ^13^C and δ^15^N [2], [17], we can visualise the ecological role of a species within their community, as its niche will incorporate all trophic interactions with prey species. Stable isotope analysis can also highlight temporal diet variability [13] and ontogenic diet shifts [18]. Delta (δ) represents the ratio of ^13^C or ^15^N relative to their lighter isotopes ^12^C and ^14^N in tissues. Carbon is conserved through trophic systems, and δ^13^C values are used to determine the source of carbon from primary producers [19], such as differentiating between oceanic (phytoplankton) and coastal (algae, seagrass and detritus) systems [15]. ^15^N is enriched through the trophic system, with consumers typically having ∼3.2‰ higher percentage mass of ^15^N than the mean value of their prey species [14], [19].

Recent studies of elasmobranch diet illustrate that tissue type greatly affects uptake and elimination rates of stable isotopes [20] and that Diet Tissue Discrimination Factors (DTDFs) cannot be assumed equal across tissues [21]. Muscle tissue shows extremely slow uptake, with over 1 year to equilibrium with diet [20] whereas metabolically active tissues such as liver are significantly quicker at both uptake and elimination of nitrogen [20]. This has a critical relevance for studies of wild populations where isotopic values in muscle tissue may not be representative of current prey species, but rather those of a spatially or temporally distinct zone (in highly migratory species). It is suggested that multi-tissue sampling produces more robust analysis of trophic position for individual species, however results may be confounded if values from different tissues (e.g. muscle and fin) are directly compared, due to different DTDFs [21].

The majority of stable isotope studies to date have used DTDFs from Post [15] (see [22] for review). However, significant variation in DTDF has been found between tissue types and species, even those of the same genus [19], so the need for species-specific validation experiments has been strongly stated [22]. To date there are only two known experimental studies of DTDFs in elasmobranchs [23], [24]. Given the long duration and logistical difficulties in conducting DTDF validation experiments on large elasmobranchs, there is an opportunity for important DTDF information to be reconstructed from stomach content data. If consumers are not in equilibrium with their diet, isotope analysis will be compromised [22], [25], however, species characterised by restricted movements within the same base isotope system (i.e. coastal or oceanic), for which prior stomach contents data exists, can be utilised to generate DTDF estimates based on SIA [22].

Southern stingrays, *Dasyatis americana*, are distributed throughout the Western Atlantic stretching from New Jersey to Brazil [26]–[30]. They are commonly found in shallow reef and lagoon habitats of coastal areas and offshore atolls, where they can reach extremely high densities [31]. *D. americana* shows very restricted movements and high site fidelity in areas characterised by sand flats, patch reefs and seagrass beds [32]. The diet of *D. americana* is relatively well characterised from six known stomach content studies [6], [10], [11], [26], [33], [34].

The simplification of marine ecosystems due to selective overfishing [35] and subsequent increases in occurrence of trophic cascades [1], [36], highlight the need of building a more robust baseline knowledge on trophic ecology of tropical marine mesopredators. Generalist mesopredators such as stingrays are of particular relevance, given their potential role in stabilizing food webs [4]; and as targeted commercial fisheries increase, the need for greater understanding of this abundant and widespread species becomes more urgent. In this study we use SIA to gain initial insight into the trophic ecology of *D. americana* using Bayesian mixing models to reconstruct diet, and Bayesian ellipse analysis to evaluate ecological niche in comparison to sympatric reef-dwelling elasmobranchs, the nurse shark *Ginglymostoma cirratum* and Caribbean reef shark, *Carcharhinus perezi*. Finally, we evaluate proposed DTDF values from the literature for use with stingrays, and propose suitable values for further work in the absence of experimental validation studies.

## Materials and Methods

All of the research (including the handling of marine life, the interruption of stingray and shark behaviour, and biopsy tissue sampling) was undertaken with research permits granted by the Belize Fisheries Department. The handling and biopsy tissue sampling of marine life complied with the Bangor University Research Ethics framework and ethical policy, and was approved by the College of Natural Sciences Animal Ethics Committee.

### Study site and animal sampling

**Figure 1:**
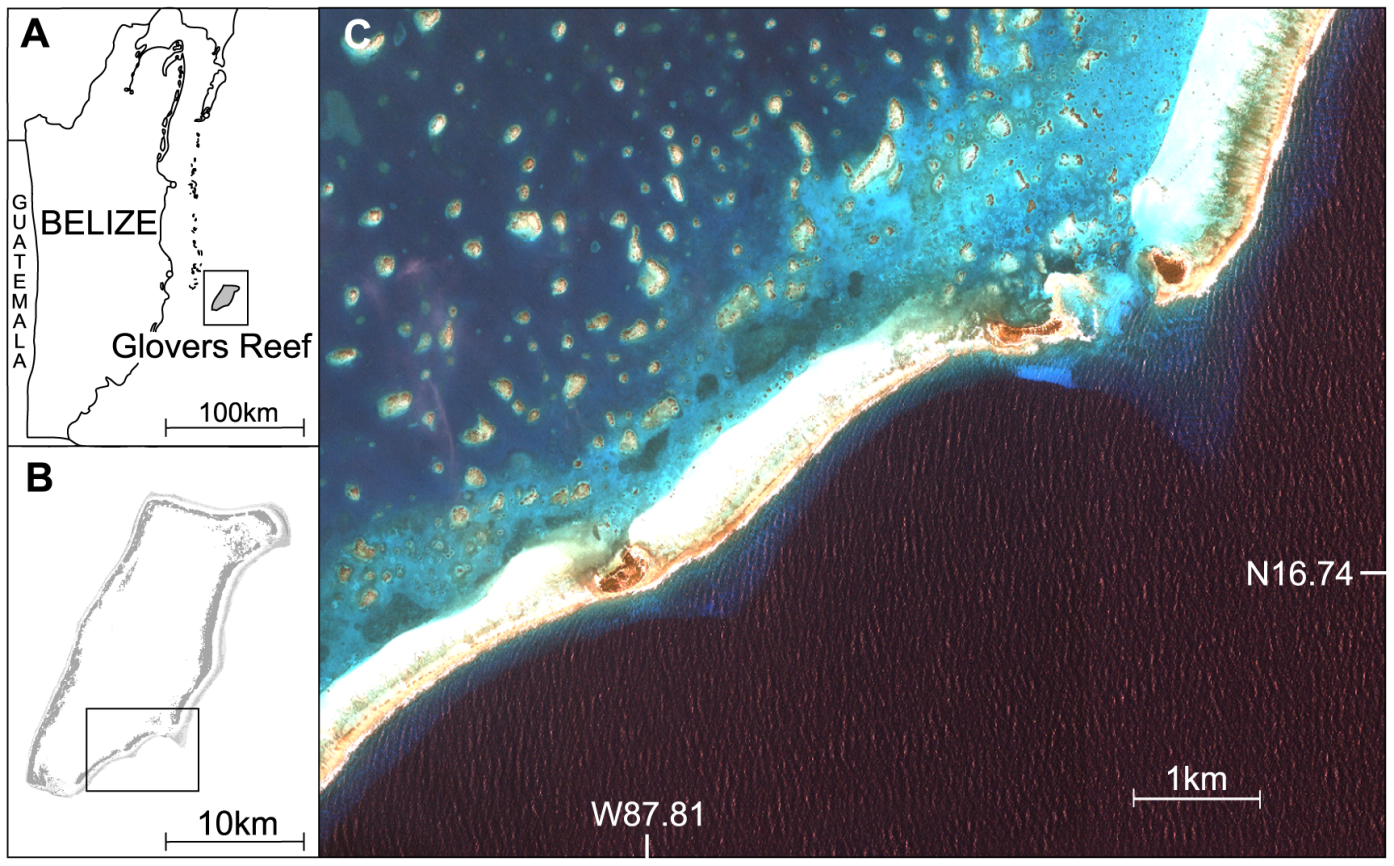
Study site location. (A) Glovers Reef Atoll within Belize, (B) Map of Glovers Reef Atoll, (C) Enlarged satellite image of the main sampling area on Glovers Reef Atoll.

Glovers Reef Atoll (GRA) (W -87.8 N 16.8) is the southernmost of four coral atolls in the Mesoamerican Barrier Reef System, situated approximately 40 km east of the Belizean coast, and 20 km east of the main barrier reef. The atoll covers approximately 254 km^2^, most of which is shallow lagoon, surrounded by a reef crest (Fig. 1).

Southern stingrays are present in the lagoon in very high densities (∼245 rays per km^2^) throughout the year [31]. Rays were caught primarily in shallow lagoon sand flat and seagrass habitat in the south east of the atoll (Fig. 1) between June 2009 and August 2010. All rays were captured individually using a gill net or long-lines in the lagoon and one by hook and line on the forereef. Rays were brought onboard and basic data was collected on sex and size (curved disk width) and capture location. Rays were biopsied using a 5 mm diameter medical muscle punch, taking a plug (∼1 g) of white muscle tissue and skin from the dorsal musculature.

Nurse sharks *G. cirratum* and Caribbean reef sharks *C. perezi* were captured using standard long lines as part of a wider research project at GRA in June 2009 and May 2010. Muscle and skin plugs (∼1 g) were taken from *C. perezi* dorsal musculature below the dorsal fin. Skin toughness of *G. cirratum* prevented muscle biopsy so only fin clips were taken from live nurse sharks for comparison with skin samples from sympatric species, to limit animal stress and handling time. One juvenile *G. cirratum* found recently dead by plastic pollution in September 2009, was opportunistically sampled for white muscle tissue during dissection, so was used as a coarse verification of fin clip tissue values.

Muscle tissue was separated from skin and sinew in all biopsy samples and all samples were stored frozen prior to drying in foil cups in a solar oven. Biopsy sampling of all sharks and rays was non-lethal and no animals were sacrificed in this study. Putative prey species (teleosts & invertebrates) identified in diet studies in the literature were collected during core sediment sampling using a vacuum pump and opportunistically during fieldwork between June 2008 and Aug 2010. Fish, conch and lobster samples were obtained from fishermen's catches with their permission.

### Trophic level

Typical diet composition of *D. americana* was obtained using four literature studies where stomach contents had been identified at least to phyla and ranked [6], [10], [11], [33].

Mean trophic levels of four prey phyla (Mollusca, Crustacea, Chordata & Annelida) were used to calculate the trophic level of *D. americana* (*TL_k_*) following Cortés [37]:
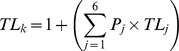
Where *P_j_* is the proportion of each prey category (*j*) in diet analyses, and *TL_j_* is their trophic level. Following analysis using stable isotope mixing models, trophic level was reassessed considering modelled diet results.

### Stable isotope ratio mass spectrometry

Animal tissue samples (∼1 mg) were analysed using automated continuous-flow isotope ratio mass spectrometry [12], [38] by the Boston University Stable Isotope Laboratory. The samples were combusted in an elemental analyser (EuroVector) and analysed using a GVI IsoPrime isotope ratio mass spectrometer (GV Instruments). Ratios of ^13^C/^12^C and ^15^N/^14^N were expressed as the relative per mil (‰) difference between the samples and international standards (Vienna PDB carbonate and N_2_ in air, respectively) where:
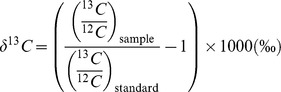
and
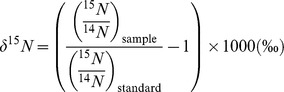
In addition to carbon and nitrogen isotopes from the same sample, continuous flow also reported % C and % N data. Urea was not removed from the muscle tissue samples for processing. Isotope ratio mass spectrometry precision was ∼0.1‰ for carbon and ∼0.2‰ for nitrogen. Lipid extraction was not carried out on sampled tissues due to mean C : N ratios being much lower than 3.5 [39].

### Multiple tissue analysis and ontogenic diet shifts

δ^13^C and δ^15^N values from *D. americana*, sharks, teleost fish and invertebrates from GRA were tested for normality, and data was analysed for differences between tissue types, species and correlation with individual size using JMP 10 (SAS Institute). Where significant differences existed between tissue types, they were treated independently in all analyses thereafter.

### Isotopic niche and prey partitioning

**Figure 2:**
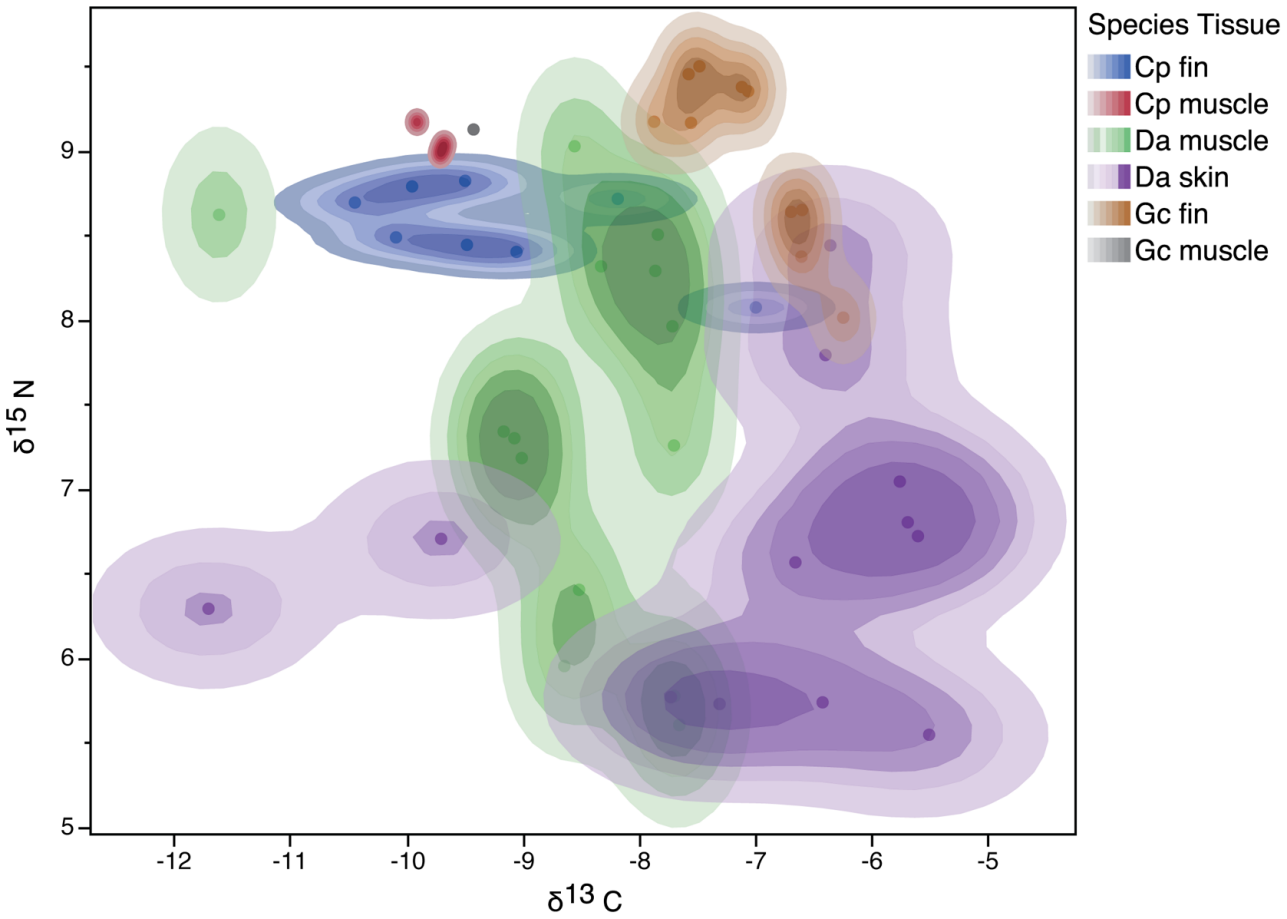
Density contour plot of δ^13^C vs. δ^15^N values for *C. perezi fin (blue) and muscle tissues* (red), *G. cirratum* fin (brown) and muscle (grey) and *D. americana* skin (purple) and muscle (green) sampled from wild populations at Glovers Reef Atoll, Belize. Different colour tones represent quartile volume contours constructed using kernel density estimation.

δ^13^C and δ^15^N values from tissues of *D. americana*, *G. cirratum* and *C. perezi* were plotted in 2D isotopic space and partitioning was visualised using kernel density estimation in JMP10 (Fig. 2).

The R package ‘Stable Isotope Bayesian Ellipses in R’ (SIBER) [17] was used to generate Bayesian ellipses of isotopic space for the three elasmobranch species. Standard Ellipse Areas (SEA) were corrected (SEAc) for low sample size using SEAc = SEA(n-1)(n-2)^−1^ [17]. Values were also analysed with Layman's metrics [40] using convex hulls of niche space, for area comparisons with Bayesian ellipses.

### Mixing models and diet-tissue discrimination factors

**Figure 3:**
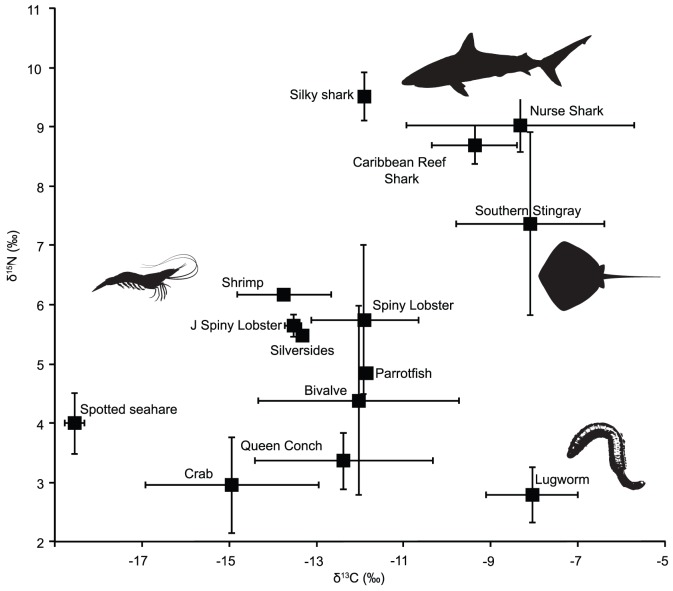
Bivariate plot of δ^13^C vs. δ^15^N values from tissue samples collected from *D. americana*, potential prey species, and sympatric shark species at Glovers Reef between 2008–2010. Values for *D. americana, G. cirratum* and *C. perezi* are displayed as combined mean (± SD) skin and muscle tissues (δ^13^C −8.09±1.7, δ^15^N 7.36±1.5; δ^13^C −8.32±2.62, δ^15^N 9.03±0.46; δ^13^C −9.37±0.98, δ^15^N 8.7±0.32 respectively).

δ^13^C and δ^15^N values from *D. americana* and benthic prey species from GRA were plotted in 2D isotopic space (Fig. 3). DTDFs from experimental and modelling studies in the literature (Table 1) were then applied to prey source values and plotted in bivariate space to analyse overlap with values from *D. americana* muscle tissue. The DTDF used from Caut et al. [19] was calculated using their equation for source values for fish. Additionally a DTDF was calculated for rays from δ^15^N values using the linear regression *y* = 5.02+0.77*x* compiled from various studies in Robbins et al. [41]. Due to small sample sizes for individual prey species, mean Δ^15^N and Δ^13^C values were calculated for each prey species and then combined into a mean value for use in mixing models.

**Table 1:** Diet-Tissue Discrimination Factors from the literature.

Label	Δ^15^N (‰)	SD	Δ^13^C (‰)	SD	Source
a)	3.39	±3.03	−0.22	±2.33	(Calculated from [19])*
b)	3.7	0.4	1.7	±0.5	[23]
c)	2.29	0.22	0.9	±0.33	[24]
d)	3.4	0.98	0.39	±1.3	[15]
e)	3.49	0.13	0.05	±0.36	[62]
f)	2.75	0.22	0.9	±0.33	[52]
g)	2.57	-	(0.39)	-	[41]

This DTDF were used to adjust prey species values of δ^13^C and δ^15^N in δ^15^N vs. δ^13^C plots, and in stable isotope mixing models of stingray diet composition. SD = Standard Deviation.

* A Δ^13^C value (or SD values) was not published in Robbins et al. [41], so the median value from all studies (0.39) was used in these plots.

δ^13^C and δ^15^N values of prey species identified from stomach contents studies in the literature [6], [10], [11], [26], [33], [34] were plotted to assess prey groupings in 2D isotopic space as prior information to enhance the accuracy of Bayesian mixing models. C : N ratios were used to evaluate differences between trophic groups for use in mixing models. Teleost fish and crab samples showed no significant difference (Wilcoxon, Z = −0.9684, p = 0.2453) so were combined as a prey category (Fish_crab) for mixing models. Similarly, shrimp and lobster values showed no significant difference so were grouped as decapods in models (Wilcoxon Z = 0.74353, p = 0.4407). The remaining 3 prey categories of bivalve, annelid and conch were significantly different, and were treated as independent groups.

The diet compositions of *D. americana* and *G. cirratum* were modelled using Stable Isotope Analysis in R (SIAR), a Bayesian stable isotope mixing model that generates probability distributions for proportions of prey items based on their relationship with consumer tissue values [42]. SIAR models were generated using δ^13^C and δ^15^N values for consumers (stingray and sharks) and putative prey items sampled from GRA, and run using DTDFs (± SD) as described above (Table 1).

## Results

Muscle and skin samples were gathered from 14 *D. americana* individuals of size range 29–77 cm disk width between June 2008 and August 2010. Eight *G. cirratum* (49–300 cm) and 9 *C. perezi* (90–200 cm) were caught in lagoon and forereef habitats and sampled for fin clips and muscle biopsies respectively.

### Trophic level

Crustaceans, predominantly crabs were proportionately the most significant prey group from gut contents studies in the literature (Table 2). Trophic level (TL) calculation for *D. americana* was considerably dependent on the TL attributed to teleost fish prey. Use of a TL value reflective of herbivorous teleost prey returned a value of 3.39, whereas inclusion of a value reflecting more carnivorous fish (∼3.4) returned a value of 3.65. Without more detailed information on species specific diet, a mean value of 2.8 from Fishbase was used for teleost fish [22], [43]. TL was calculated to be 3.52 (±0.31) for *D. americana*, corroborating the figure reported for this species in Fishbase [43]. Variation in the proportion of annelid and hemichordate prey (TL ∼2.5) in diet, as between findings of Gilliam & Sullivan [11] and Randall [10], had no influence on trophic level, likely due to the proportion of crustaceans at similar trophic level placement (TL ∼2.52).

**Table 2:** Major prey identified from stomach analysis studies of *D. americana*, according to lowest taxonomic group (as stated in source).

Source	Location	N	Prey group
[11]	Exuma Cays Land and Sea Park, The Bahamas	18	46% Decapods & Portunids
			18% Teleosts
			7% Stomatopods
[33]	Indian River Lagoon, Florida	3	Portunids
			Caridea
			Teleosts
[10]	Puerto Rico & Virgin Islands	25	38% Sipunculids/Polychaetes
			22% Teleosts
			18% Crabs
			11% Bivalves
			8% Shrimps
[34]	Florida Bay, Florida	5	Decapods
			Stomatopods
			Portunids
			Caridea
[6]	Cape Hatteras, North Carolina	2	99% Decapods
			<1% Teleosts
			<1% Polychaetes
[26]	Bimini, The Bahamas	15	Stomatopods
			Shrimps
			Crabs
			Worms
			Fish

Prey groups are listed in order of decreasing proportional volume where data available. Unnumbered prey indicates prey proportions were not ranked. Column N represents the number of rays sampled in each study.

### Multiple tissue analysis and ontogenetic shift

δ^13^C values showed greater intra-species variation in elasmobranch species sampled than δ^15^N values. Mean δ^15^N values from *D. americana* were not significantly different between skin (6.6‰±0.9) and white muscle tissue (7.4‰±1.1) (t-test t(24) = P<0.06), however, mean δ^13^C values for skin (−7.1‰±1.9) were significantly higher than those for white muscle (−8.5‰±1.0) (t(24) = 2.49, P = 0.02).

**Figure 4:**
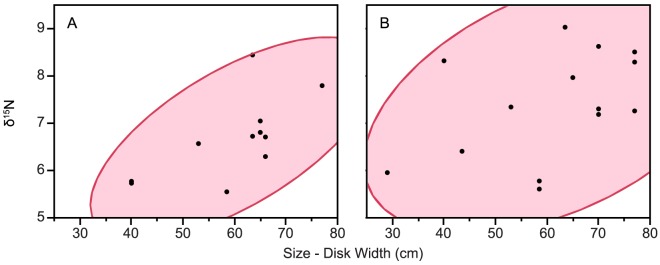
Relationship between δ^15^N values and individual size (disk width) for *D. americana* skin (A) and muscle (B). Red shaded areas represent 95% bivariate normal confidence ellipses for correlative relationships (A) Spearman's r(8) = 0.75 P<0.034 (B) Spearman's r(11) = 0.47, P = 0.15.

δ^15^N values in *D. americana* skin tissue showed a significant positive tendency with individual size, (Spearman's r(8) = 0.62 P = 0.01) (Fig. 4A) however the relationship with muscle tissue was non-significant (Spearman's r(11) = 0.36, P = 0.28) (Fig. 4B).

The mean (± SD) C : N ratio for *D. americana* muscle tissue was 1.12‰±0.27; *G. cirratum* fin tissue was 0.81‰±0.079; and *C. perezi* muscle tissue was 1.08‰±0.098.

δ^15^N and δ^13^C values were unaffected by calendar month sampled (ANOVA F = 1.72, df = 7, P = 0.26 and ANOVA F = 2.22, df = 7, P = 0.16 respectively) however, not all calendar months were sampled.

The mean δ^15^N (± SD) value of skin tissue was 6.6‰±0.9 for *D. americana* and 9.03‰±0.46 for *G. cirratum* illustrating a significant difference (Wilcoxon Z = 4.66 P<0.001). Mean δ^13^C values showed no significant difference (*D. americana* −8.32‰±2.62; *G. cirratum* −8.53‰±1.12; Wilcoxon Z = 1.57 P = 0.22).

### Isotopic niche and prey partitioning

**Figure 5:**
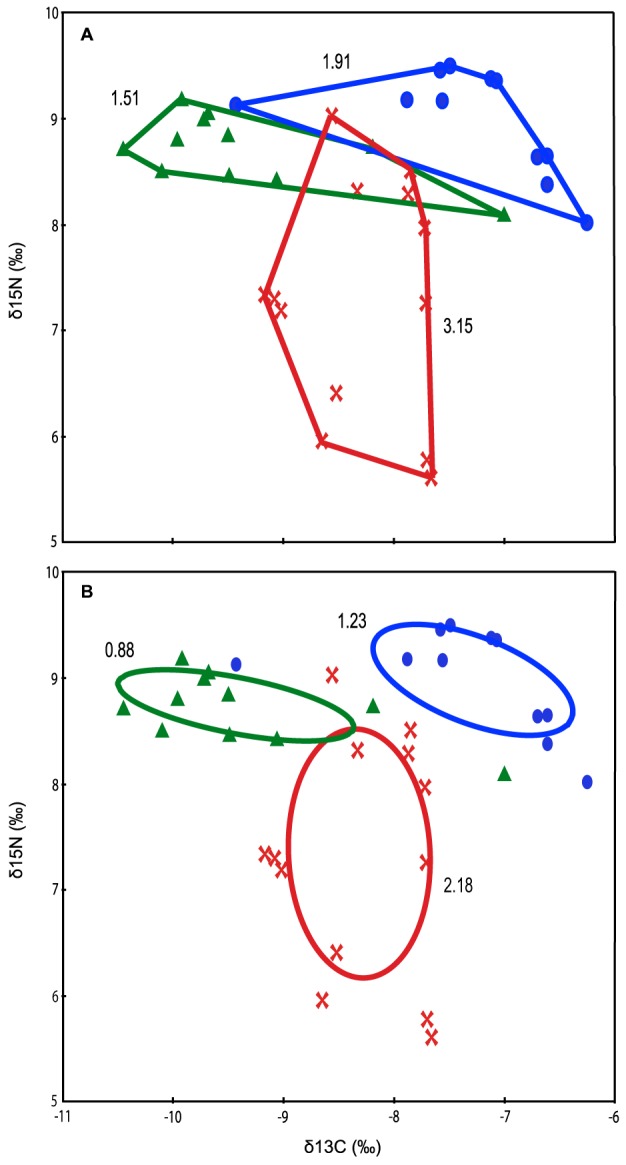
Bivariate plots of isotopic space depicting niche areas for muscle tissue of *Dasyatis americana* (red), *Ginglymostoma cirratum* (blue) and *Carcharhinus perezi* (green), using (A) convex hull areas [40], and (B) Bayesian ellipses [17].

For muscle tissue only, isotopic niche space of *D. americana* was larger than sympatric species using both analytical measures of area calculation. Convex hulls presented a niche space for southern stingrays of 3.15, with 1.91 and 1.51 for nurse sharks and reef sharks respectively (Fig. 5A). Corrected Bayesian ellipse areas calculated *D. americana* niche space as 2.18 and *G. cirratum* and *C. perezi* as 0.88 and 1.23 respectively (Fig. 5B). *D. americana* exhibited a much wider range of δ^15^N values (3.42‰) than *G. cirratum* (1.48‰) and *C. perezi* (1.09‰). The opposite was true for the range of δ^13^C values, with *G. cirratum* (3.18‰) and *C. perezi* (3.45‰) exhibiting higher values than *D. americana* (1.51‰).

### Mixing models and Diet-Tissue Discrimination Factors

As expected, the different DTDFs used in bivariate plots and Bayesian modelling with SIAR produced various results for diet composition. Bivalves and lugworms were consistently represented as a high proportion of prey for *D. americana* for all DTDFs analysed. The lowest magnitude of DTDF [24] influenced the emergence of shrimp as an important dietary item, whereas the highest experimental DTDF value [23] increased the probability of higher proportions of conch (*Strombus gigas*) in stingray diet. All DTDF values utilised in combined mixing models returned results suggesting smaller median contributions of crab (and teleost fish) to stingray diet than suggested by stomach contents studies.

## Discussion

This study is an important first step towards a greater understanding of the ecological niche and function of an epibenthic generalist, that can reach extremely high abundances in tropical marine communities [31]. The trophic level of 3.52 (±0.31) we calculated for *D. americana* from published stomach contents is lower than the mean value of 3.65 for chondrichthyan fish [37], and places *D. americana* at the lower end of the genus *Dasyatidae* in terms of trophic level, implying that Caribbean rays may occupy a niche lower on the food chain than elsewhere. The generalist, opportunistic diet of *D. americana* [11], [26] would imply a certain fluctuation of trophic level according to geographic location and variation in prey availability, and the wide isotopic niche seen in stingrays at GRA may further suggest a large degree of individual variation in trophic level, as found with *Dasyatis lata* in Hawaii where trophic level varied from 3.2 to 4.5 with individual size [44]. This variation may relate to ontogenetic diet shifts [45], [46], where growing predators feed on progressively larger prey items, or a completely distinct prey set [47]. Isotopic nitrogen values in stingray muscle and skin tissues showed positive correlations with size, however, stingrays tracked acoustically at GRA showed no expansion of activity space or habitat shifts with ontogeny [32]. This suggests that an increase in δ^15^N could be attributed to eating larger prey items rather than a diet shift, however greater sampling of consistent tissue type will be needed to confirm positive ontogenetic diet shifts. In some cases, changes in δ^15^N levels can be attributed to a geographical shift to systems of varying base nitrogen level [13], [44]. Levels of δ^13^C in stingray tissues and their prey species at GRA imply they are reliant upon a seagrass system with base δ^13^C values of ∼8–13‰ [48]. Any movement to feed off the atoll would result in lower carbon baseline values, characteristic of an ‘offshore’ system of phytoplanktonic primary producers [13], and there is no evidence to suggest that any stingray movement or life stage is associated with a pelagic environment, so this is unlikely to explain the ontogenetic variation seen in δ^15^N values at GRA. The two stingrays caught on the forereef of GRA showed marginally lower δ^13^C values than rays caught in the lagoon, but due to low stingray frequency and sampling difficulties associated with this habitat when compared to the lagoon, sample size was considered too small to make conclusive inferences.

Our results from mixing models suggest that the diet of *D. americana* is dominated by soft-bodied invertebrates (annelid & polychaete worms) and bivalves, fitting closely with the findings of Randall [10] who also ranked annelids and hemichordates as the largest contributors to diet. The low importance of crabs and teleosts in mixing models was unexpected given their prevalence in more recent stomach contents studies [6], [11]. This may be explained by an underrepresentation of soft-bodied, rapidly-digested prey such as annelids & polychaetes in previous gut contents studies, or conversely an over-representation of hard shelled prey items [49]. Bivalves (*Tellina* spp., *Iphigenia brasiliana*) were also prominent in model results, second to annelids. Randall [10] suggests that low frequencies of shells in gut contents are a consequence of rays selecting only soft tissue from crushed molluscs. Work with cownose rays supports this, where ingestion of entire shells occurs only with thin-shelled prey, whereas thick shells are pried from tissue before consumption [50].

The larger proportion of burrowing worms in mixing models may suggest these are a consistent prey item, consumed regularly and in quantity, whereas crustaceans and teleosts are more opportunistic prey (where capture may be of less energetic effort when they are found). Furthermore, the relative importance of these soft-bodied burrowing prey may imply that the ability to sense prey effectively and excavate to sufficient depth are more crucial to foraging success, and thus would increase with ontogeny through learning and experience. Gilliam & Sullivan [11] report *D. americana* consuming at least 65 prey types, and as many as 30 items per individual throughout the day [11], further supporting a fairly continuous and opportunistic feeding strategy. Three out of the four diet studies using ranked stomach contents for *D. americana* showed diet to be predominated by crustaceans and teleosts, however evidence of *D. americana* feeding exclusively on lancelets (*Branchiostoma floridae*) in Florida [51] suggests that local environmental variability in prey abundance is a strong driver in dietary choice for such a generalist mesopredator.

The only known study of stingray trophic position utilised an assumed DTDF of 2.7‰ [44], equating to that proposed by Vanderklift & Ponsard [52]. In mixing models, all values of DTDF (2.29–3.7‰) supported the primary importance of bivalves and annelids in stingray diet at GRA. Recent studies suggest that the magnitude of DTDF relates to the nitrogen properties of the predator diet, where prey items containing a high percentage of quality protein, will result in smaller DTDFs [19], [41], [53]. The predominantly invertebrate diet of *D. americana* represents a source of relatively low quality protein compared to large sharks on a proteinaceous diet. Experimental feeding studies on sharks have shown highly variable DTDFs for nitrogen ranging from 2.2‰ [24] to 3.7‰ [23], a wide range that could significantly bias or confound ecological interpretations. Our bivariate plots and mixing models suggest that a DTDF for nitrogen in *D. americana* muscle tissue would not exceed 3‰, and that the DTDF for carbon in muscle tissue is relatively high at ∼1‰. Additional information on stingray diet studies from the literature support our findings and make the Δ^15^N ≊ 2.7‰ and Δ^13^C ≊ 0.9‰ DTDF values suggested by Vanderklift & Ponsard [52] the most appropriate; fitting closely to regression lines in Robbins et al. (2010); and also corroborating results for brown stingrays, *D. lata*, using this same value [44]. Lipid extraction is often performed to standardise SIA across species and tissue types [54]. However, mean C : N ratios of stingray and shark tissues were well below values recommended for correction or extraction (<3.5) [15], [55] and showed very little variance, so this is unlikely to have affected our results.

A diverse prey base is supported by isotopic niche analysis using both Layman's metrics and Bayesian ellipses, where southern stingrays exhibited a much greater trophic niche than *G. cirratum* and *C. perezi*, indicating large individual variation characterised by a generalist diet with a considerable number of prey interactions on different trophic levels. Conversely, *C. perezi* exhibited a very restricted niche implying more specific proteinaceous diet preferences as we might expect, likely to be characterised by a few strong interactions [5]. *G. cirratum* values indicated it feeds at the same trophic level as *C. perezi*, but the little overlap of δ^13^C value ranges represented may suggest effective prey resource partitioning between these sympatric reef dwelling shark species, however further sampling focused on these species is needed. Diet studies on *G. cirratum* suggest they prey on squids, shrimps, crabs, spiny lobsters, sea urchins and predominantly herbivorous fishes [10], [26], [56], accounting for the larger trophic niche than *C. perezi*. The location of both shark isotopic niches at higher values of δ^15^N is indicative of feeding at a consistently higher trophic level than *D. americana*. Isotopic space from Bayesian ellipses, in correcting for small sample size, provide further evidence to suggest that very little prey partitioning occurs with sympatric shark species, but rather nurse sharks and stingrays appear to feed at different trophic levels (Fig. 5). Greater sampling would allow for stronger inferences to be made, however this finding provides further insight into the potential importance of stingrays as omnivores, and highlights the need for further work on their role in stabilising lower levels of the foodweb to trophic cascade, following removal of apex predators.

The diversity of prey items consumed by stingrays as opportunistic generalists may complicate and confound the use of SIA in studying diet [22], where location and sampling season may cause significant variations as seen in the common stingray *Dasyatis pastinaca* in the Black sea [49]. This would suggest that diet variations recorded in *D. americana* are responses to different sampling period, however the Glovers Reef lagoon is a relatively stable environment with low flushing rates, limited wave action and stable sediments, and no variation with sampling month was observed at GRA within the sampled months. Given the high site fidelity of stingrays and restricted movement within the same base isotope system [32], stark seasonal variations to diet are unlikely, however, more complete seasonal sampling will be necessary to more confidently assess this.

Natural variation between sampling regions is likely to account for some dietary differences, where rays have been shown to respond to: environmental and temporal variations in prey communities and abundances [51], [57]; differing substrate complexity and prey availability [58]; or the response to ecological forces of predation and competition controlling the foraging intensity of rays in different habitats. Without combined sampling of both stomach contents and SIA, the reasons for these differences are speculative, however, SIA provides a metric for trophic niche of sympatric species, and important insight into the ecological role of mesopredators in marine benthic communities.

The different isotopic characteristics seen in skin and muscle tissues from stingrays highlight the need for sampling consistency, yet may also represent important ecological information as skin exhibit significantly higher levels of δ^13^C, and would be expected to have a faster isotopic turnover rate than muscle [21]. Further analysis using different tissue types from rays may allow for comparison of long and short-term dietary preferences or temporal shifts. In addition, given the limited sampling of stingrays larger than the documented female maturity size of ∼70 cm [59], further sampling of large rays will be needed to confirm ontogenetic diet trends. If positive relationships seen between individual size and ^15^N continue to increase with ontogeny, this may suggest possible resource competition between large rays and sympatric nurse sharks illustrated by overlapping niches in stingray upper trophic range.

The small sample size of our study limits the resolution of our inference of prey choice and trophic dynamics between sympatric elasmobranchs, however given the growing body of evidence pointing to the importance of omnivores in stabilising marine communities to trophic cascade [4], [60] especially as a consequence of selective fishing pressure [5], our study highlights the need for more information on the trophic ecology of generalist mesopredators. Furthermore, the increased targeting of large-bodied mesopredators in developing nation fisheries [28], [30], [61] adds additional urgency in discovering the knock on effects of their removal from marine communities. This study provides important preliminary information on stingray diet and trophic niche, on which to base further work and conservation action.
